# Characteristics of patients with frequent contact with general practice: a retrospective study

**DOI:** 10.3399/BJGPO.2024.0195

**Published:** 2025-07-30

**Authors:** Jesper B Nielsen, Helene S Andersen

**Affiliations:** 1 Research Unit for General Practice, Department of Public Health, University of Southern Denmark, Odense, Denmark

**Keywords:** general practice, frequent attender, primary health care, Denmark

## Abstract

**Background:**

Frequent attenders (>11 annual contacts) use more resources than most other patients in general practice.

**Aim:**

To study what characterises frequent attenders in relation to age, sex, mode of contact (face to face, email, or phone), and patient contact (GP or GP staff).

**Design & setting:**

A retrospective analysis of Danish patient datasets in general practice.

**Method:**

We used patient data from 11 Danish GP clinics and 38 874 patients covering a 12-month period. Bivariate as well as regression analyses of patient data were used.

**Results:**

Frequent attenders exist in all age groups, but with different frequencies. In the age group 55–64 years, 25% of patients were frequent attenders, this increased to 33% in the next age decade from age 65–74 years and reached 48% among those aged ≥75 years. Frequent attenders have a different user pattern related to physical visits, phone consultations, or email consultations than other patients. In their contact to the GP clinic, the frequent attender is relatively more often in contact with the practice staff than a GP compared with other patients.

**Conclusion:**

Within our Danish patient population, 22% had >11 annual contacts to their GP clinic. These frequent attenders are in general terms characterised by being female, aged >65 years, having 20 annual contacts to the GP clinic, and having ≥10 diagnoses. For the entire patient population, the GP handles 36% of the patient contacts (64% by other staff members), and this percentage is lowest among frequent attenders. Email contact is used more often among frequent attenders than other patients.

## How this fits in

Frequent attenders (>11 annual contacts) use more resources than most other patients in general practice, and GPs are challenged by increasing workload. Therefore, it is pertinent to study what characterises this group of patients in relation to age, sex, mode of contact (face to face, email, or phone), and patient contact (GP or GP staff). Frequent attenders are in general terms characterised by being female, aged >65 years, having 20 annual contacts to the GP clinic, and having ≥10 diagnoses. In our study population GPs handle 36% of the patient contacts (64% by other staff members), and this percentage is lowest among frequent attenders.

## Introduction

Some people contact their GP seldom, others many times annually. In the UK, the number of consultations with a GP attributed to frequent attenders has increased over time from a median of 13 annual consultations in 2000 to 21 in 2019, and consequently they progressively contribute to an increased workload.^1^ It is therefore pertinent to study and compare what characterises those patients who very frequently contact their GP and those who do not.

The existing literature on frequent attenders is a combination of cross-sectional cohort studies, follow-up studies, register studies, and qualitative studies based on patient interviews. Size-wise, studies span from interviews of 75 patients,^2^ to cohort studies with >50 000 patients;^3^ a follow-up study including around 1.8 million visits,^4^ to a retrospective cohort study including >12 million patients.^1^ The different methodological approaches often offer different answers, which are all related to frequent attenders.

Frequent attenders are most often defined by a certain proportion of a patient population, for example, the 10% with most frequent practice contact, or by the frequency of contacts, for example,>10 annual contacts.^5^ Further, some studies include only the physical visit at the GP’s office, whereas others also include home visits, telephone consultations, email consultations, and video consultations. Moreover, an increasing proportion of consultations are handled by trained nurses, which is not always clear from registers. These differences challenge comparisons between studies.

A recent Danish register study found that among patients aged >75 years women more frequently than men had phone consultations (Olsen J *et al*, unpublished data, 2025). Moreover, they reported that the distribution of the reported 10 annual contacts between physical visits in the GP clinic, phone consultations, email consultations, and home visits was 37%, 33%, 22%, and 6%, respectively. Video consultations comprised less than 1% of consultations.

Generally, existing studies point at specific disease groups, age, and socioeconomic factors as individual determinants. Thus, and not unexpected, the frequent attender group is associated with higher medical need for care,^6^ drug use and polypharmacy,^7^ but also associated with more subjective health-related factors.^8^ Low global functioning (Global Assessment of Functioning [GAF] score)^2^ and unemployment^3,6^ are among the less well-defined factors associated with frequent attenders. Age appears to be a bit ambiguous with studies pointing towards both high and lower age as independent factors (Olsen J *et al*, unpublished data, 2025),^6,9^ whereas agreement exists that females are more common among frequent attenders than males.^3,10^ Continuity of care is seen as a positive asset in primary care settings, and it is therefore interesting that a recent cohort study from UK did not find any association between continuity in general practice and frequent attendance.^11^

The demand from patients to contact general practice has increased over the recent decades. During the same period, and with the purpose of reducing the workload on the individual practitioner, other contact types, such as email, have emerged and many tasks, for example, annual controls, have been transferred from the GP to clinical staff. As the frequent attenders are an increasing proportion of the patient population, it appears relevant also to study to what extent the user pattern of general practice is different between frequent attenders and the remaining patient population considering both patient characteristics and user patterns in general practice.

The present study is based on full access to GP files on 38 874 patients from 11 Danish GP clinics covering a full 12-month period. The sample size and age span allowed for subgroup analyses complementing previous research, and presents analyses on the influence of age, sex, clinical staff, frequency of contacts, and consultation type.

The overall aim of the study is to improve the available knowledge base on factors associated with frequent attenders for discussions and decisions related to access policies to GP clinics and how best to meet the need of all patients.

## Method

In Denmark, general practice may be made up of private companies that are owned by one or a few GPs or it may be organised with a structure consisting of a central administration owning and running several GP clinics each with individually hired GPs. The data used in this study are based on activities at Alles Lægehus (a central administrative unit responsible for running >30 individual GP clinics) and consisted of two different datasets from those clinics that were part of Alles Lægehus for the entire 1-year period. The first dataset had a list of registered patients at the GP clinics administered by Alles Lægehus from January 2019 and April, May, November, and December 2020. The second dataset contained an overview of dates of patient contacts between 1 February 2019 and 1 April 2021.

In both datasets all patients could be identified by their personal identification number (CPR), which is a unique number given to all Danish citizens, where one’s birthday can be derived. Along with the date of contact, information was also included on sex, age at contact, if the person had been in contact with a GP or other personnel at the unit, the form of consultation (physical attendance, online, phone, or email), and if one is given, a diagnosis following the contact.

We limited the timeframe to 1 February 2019 to 31 January 2020. This timeframe was chosen so that the contacts were before Denmark was hit by restrictions to COVID-19 in March 2020.

Further, our data was restricted using the following criteria:

- only clinics providing a list of registered patients and dates of contacts;
- only contacts in the relevant time interval; and
- only patients with at least one contact within the time interval.

As it was not possible to assure that zero contacts were not owing to patients who had changed to another GP or clinic, we excluded patients with zero contacts from our regression analyses.

Patients were split into three groups based on number of contacts for each interval, 1–2 contacts, 3–11 contacts, and >11 contacts, not differentiating between mode of contact or personnel. For reasons of comparability between studies, we chose absolute numbers to differentiate the groups instead of percentages. To minimise the risk of double-counting patients getting same-day response, only one contact a day with the same personnel group or mode of contact was allowed.

To study the relation between number of contacts, sex, age, and number of diagnoses, we used a multinomial logistic regression model, where we considered intragroup correlation of clusters based on the units. Results were considered statistically significant for *P*-values <0.05. Stata (version 18) was used for all analyses.

## Results

**Figure 1.**
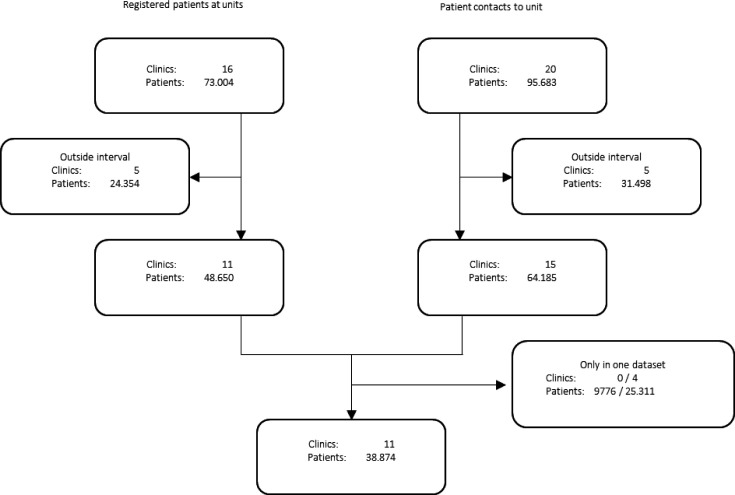
Flowchart on registered patients and contacts

Eleven GP clinics with 38 874 enlisted patients were included. The criteria for inclusion were that data on registered patients as well as contacts should cover the entire 1-year period (Figure 1). Five clinics were excluded as they did not provide patient lists for the entire 1-year period, and five clinics were excluded as they did not provide data for patient contacts for the entire 1-year period. Eventually, clinics and patients not present in both datasets were excluded.

The number of patients excluded from data analyses on the 11 clinics because they were without contact to their GP during the 1-year periods was 9776 (9776/48 650 = 20.1% of patients).

Among patients with contacts to their GP clinic within the 1-year period, 9931 (25.5%) of patients contacted their GP clinic 1–2 times, 20 379 (52.4%) 3–11 times, and 8564 (22.0%) >11 times (Table 1). In overall terms, 10% of the patients were responsible for about 30% of all contacts (data not shown). Females constituted 51.4% of the study population but 61.6% of the frequent attenders. The mean age was highest among frequent attenders, and the proportion of frequent attenders increased with age reaching just over one in four aged ≥75 years (Table 1). Also, 30.7% of frequent attenders had ≥10 registered diagnoses, and frequent attenders comprised 95.9% of patients with ≥10 registered diagnoses (Table 1). In the age group 55–64 years, 25.0% of patients were frequent attenders, this increased to 33.2% in the next age decade and increased to 47.8% among those aged ≥75 years (calculated from Table 1).

**Table 1.** Baseline characteristics for the population for each interval, *n* (%)

	1–2 contacts	3–11 contacts	>11 contacts	All	*P*-value
**Participant**	9931 (100.0)	20 379 (100.0)	8564 (100.0)	38 874 (100.0)	
**Sex**					<0.001
Female	4156 (41.8)	10 540 (51.7)	5275 (61.6)	19 971 (51.4)	
Male	5775 (58.2)	9839 (48.3)	3289 (38.4)	18 903 (48.6)	
**Age, years**					<0.001
Mean	35.0	44.6	58.6	45.2	
0–14	2303 (23.2)	2313 (11.3)	134 (1.6)	4750 (12.2)	
15–24	1577 (15.9)	2613 (12.8)	527 (6.2)	4717 (12.1)	
25–34	1410 (14.2)	2589 (12.7)	795 (9.3)	4794 (12.3)	
35–44	1167 (11.8)	2223 (10.9)	737 (8.6)	4127 (10.6)	
45–54	1205 (12.1)	2759 (13.5)	1031 (12.0)	4995 (12.8)	
55–64	1124 (11.3)	3174 (15.6)	1429 (16.7)	5727 (14.7)	
65–74	737 (7.4)	2717 (13.3)	1713 (20.0)	5167 (13.3)	
≥75	408 (4.1)	1991 (9.8)	2198 (25.7)	4597 (11.8)	
**Diagnoses**					<0.001
Mean	0.7	2.8	8.2	3.4	
No diagnoses	4444 (44.7)	2036 (10.0)	126 (1.5)	6606 (17.0)	
1–4 diagnoses	5487 (55.3)	14 799 (72.6)	1981 (23.1)	22 267 (57.3)	
5–9 diagnoses		3431 (16.8)	3825 (44.7)	7256 (18.7)	
10–19 diagnoses		113 (0.6)	2302 (26.9)	2415 (6.2)	
20–39 diagnoses			303 (3.5)	303 (0.8)	
≥40 diagnoses			27 (0.3)	27 (0.1)	

The 11 GP clinics had in total 306 845 contacts (Table 2), and among those in contact with their GP clinic, the average frequency of contacts was 7.9 contacts per enlisted patient-year. The 8564 frequent attenders contacted on average their GP clinic 19.7 times within the 1-year period (Tables 1 and 2). Regarding point of contact, 35.7% of all contacts were handled by a GP, slightly more (41.0%) for patients with only 1–2 annual contacts and slightly less (33.3%) for patients with >11 annual contacts (Table 2). Thus, a larger proportion of the patients with frequent attendance were handled by the clinical staff and not the GP. And visa versa for the small group of patients seen only a few times annually in the clinic.

**Table 2.** Point of contact and modes of contact by number of annual contacts for each interval, *n* (%)

	1–2 contacts	3–11 contacts	>11 contacts	All
**All**	14 483 (100.0)	123 401 (100.0)	168 961 (100.0)	306 845 (100.0)
**Point of contact**				
Personnel	8545 (59.0)	76 075 (61.6)	112 730 (66.7)	197 350 (64.3)
GP	5938 (41.0)	47 326 (38.4)	56 231 (33.3)	109 495 (35.7)
**Mode of contact**				
Physical attendance	8770 (60.6)	71 271 (57.8)	88 318 (52.3)	168 359 (54.9)
Email	1668 (11.5)	13 376 (10.8)	28 115 (16.6)	43 159 (14.1)
Phone	4045 (27.9)	38 754 (31.4)	52 528 (31.1)	95 327 (31.1)

Regarding mode of contact, 54.9% of all contacts were physical visits at the clinic, 14.1% email consultations, and 31.1% were contacts through telephone (Table 2). Video consultations were below 0.1% of contacts and could not be analysed separately. Deviating from this general picture were patients with 1–2 annual contacts, where contacts were more often by physical visits (60.6%) and less often by phone (27.9%). Further, patients with >11 annual contacts had a slightly different balance between phone, email, and physical consultations (more email, less physical visits) than patients with 3–11 annual contacts.

Males had 58% lower risk having >11 contacts annually than females (relative risk ratio [RRR]: 0.42). (Table 3).

**Table 3.** Multinomial logistic regression for contacts to general practice allowing for intragroup correlation between units and adjusted for sex and age

		RRR	95% CI	*P*-value
**3–11**				
	Sex (Male)	0.65	(0.62 to 0.69)	<0.001
	Age, years			
	0–14	0.35	(0.30 to 0.40)	<0.001
	15–24	0.57	(0.49 to 0.67)	<0.001
	25–34	0.64	(0.58 to 0.71)	<0.001
	35–44	0.66	(0.61 to 0.73)	<0.001
	45–54	0.80	(0.72 to 0.89)	<0.001
	55–64 (Ref)	Ref	Ref	Ref
	65–74	1.32	(1.14 to 1.52)	<0.001
	≥75	1.71	(1.49 to 1.97)	<0.001
**>11**				
	Sex (Male)	0.42	(0.39 to 0.45)	<0.001
	Age, years			
	0–14	0.04	(0.03 to 0.06)	<0.001
	15–24	0.25	(0.19 to 0.33)	<0.001
	25–34	0.43	(0.35 to 0.53)	<0.001
	35–44	0.48	(0.43 to 0.54)	<0.001
	45–54	0.66	(0.61 to 0.71)	<0.001
	55–64 (Ref)	Ref	Ref	Ref
	65–74	1.86	(1.56 to 2.22)	<0.001
	≥75	4.15	(3.46 to 4.98)	<0.001

CI = confidence interval. RRR = relative risk ratio.

The age group 55–64 years was the largest group and was chosen as reference group for the regression analyses related to age. Being aged 0–14 years makes the risk of having 3–11 contacts 65% lower than the 55–64-year-old group after adjusting for sex and mean numbers of diagnosis. For aged ≥75 years, there is a 71% increased risk of having 3–11 annual contacts compared with the 55–64-year-old group after adjustments (Table 3). It is worth mentioning that among children (aged <15 years) and in the age group ≥74 years, there was no significant sex difference in the relative occurrence of frequent attenders (data not shown). Also, the risk of having >11 annual contacts among people aged ≥65 years is significantly higher (186–415%) than for people aged 55–64 years (Table 3). Thus, female sex and age appears to be strong predictors of having frequent contacts with the GP clinic.

In general, for patients aged 15–74 years age does not influence the balance between type of contact (phone, email, physical GP visit) much (Table 4). However, for children (aged 0–14 years) a higher proportion of visits were physical and fewer contacts handled by email (Table 4), and for patients aged ≥74 years there was a decrease in the proportion of physical visits and phone consultations, but an increased proportion of contacts being handled by email. We are not able to differentiate between email correspondence taken care of by the patient or by relatives or nursing home staff.

**Table 4.** Contact mode by age of patient, *n* (%)

	Phone	Email	Physical	Total
0–14	5446 (32.25)	509 (3.01)	10 934 (64.74)	16 889 (100)
15–24	8509 (32.53)	2767 (10.58)	14 884 (56.90)	26 160 (100)
25–34	10 058 (32.11)	4219 (13.47)	17 050 (54.43)	31 327 (100)
35–44	9124 (31.99)	3604 (12.64)	15 789 (55.37)	28 517 (100)
45–54	12 727 (32.73)	5030 (12.93)	21 133 (54.34)	38 890 (100)
55–64	15 104 (31.12)	6660 (13.72)	26 768 (55.16)	48 532 (100)
65–74	16 484 (30.47)	7216 (13.34)	30 406 (56.20)	54 106 (100)
≥75	17 875 (28.63)	13 154 (21.07)	31 395 (50.29)	62 424 (100)

The adjusted multinomial logistic regression analysis for contacts to general practice stratified for point of contact (GP or other clinical staff) confirmed that the proportion of females increased with increased frequence of attendance, and irrespectively of whether the contact was with a GP or other clinical staff (Table 5).

**Table 5.** Multinomial logistic regression allowing for intragroup correlation between units and adjusted for sex and age and stratified for point of contact

		Personnel			GP		
		RRR	95% CI	*P*-value	RRR	95% CI	*P*-value
**3–11**							
	Sex (Male)	0.69	(0.66 to 0.74)	<0.001	0.59	(0.57 to 0.62)	<0.001
	Age, years						
	0–14	0.34	(0.29 to 0.39)	<0.001	0.37	(0.31 to 0.45)	<0.001
	15–24	0.57	(0.49 to 0.67)	<0.001	0.56	(0.45 to 0.68)	<0.001
	25–34	0.64	(0.54 to 0.75)	<0.001	0.65	(0.57 to 0.73)	<0.001
	35–44	0.67	(0.59 to 0.75)	<0.001	0.64	(0.58 to 0.70)	<0.001
	45–54	0.80	(0.70 to 0.90)	<0.001	0.78	(0.67 to 0.90)	<0.001
	55–64 (Ref)	Ref	Ref	Ref	Ref	Ref	Ref
	65–74	1.25	(1.09 to 1.44)	0.001429	1.41	(1.24 to 1.60)	<0.001
	≥75	1.58	(1.43 to 1.74)	<0.001	1.99	(1.70 to 2.34)	<0.001
**>11**							
	Sex (Male)	0.45	(0.41 to 0.48)	<0.001	0.38	(0.34 to 0.42)	<0.001
	Age, years						
	0–14	0.04	(0.03 to 0.06)	<0.001	0.05	(0.04 to 0.06)	<0.001
	15–24	0.25	(0.20 to 0.32)	<0.001	0.25	(0.18 to 0.35)	<0.001
	25–34	0.43	(0.33 to 0.55)	<0.001	0.44	(0.36 to 0.54)	<0.001
	35–44	0.50	(0.45 to 0.55)	<0.001	0.47	(0.41 to 0.54)	<0.001
	45–54	0.67	(0.62 to 0.73)	<0.001	0.63	(0.54 to 0.73)	<0.001
	55–64 (Ref)	Ref	Ref	Ref	Ref	Ref	Ref
	65–74	1.78	(1.50 to 2.10)	<0.001	2.03	(1.68 to 2.45)	<0.001
	≥75	3.84	(3.22 to 4.57)	<0.001	5.02	(4.15 to 6.07)	<0.001

CI = confidence interval. RRR = relative risk ratio.

## Discussion

### Summary

Generalising our data, a frequent attender is a female, is aged >65 years, has around 20 annual contacts to the GP clinic, and has ≥10 diagnoses. Frequent attenders (and older patients) have a different user pattern related to physical visits, phone consultations, or email consultations than other patients as they have relatively fewer physical and phone consultations, but more email contacts. In their contact to the GP clinic, the frequent attender is more often in contact with the practice staff than the GP. This makes sense as these contacts are more often follow-up consultations (controls) and annual check-ups and liberates more time for the GP to handle children and patients not so well-known to the clinic.

### Strengths and limitations

A sample the size of 38 874 patients from 11 different GP practices is a strength, which allows for robust and subgroup analyses. Further, it is a strength that we have a full and identical 12-month sampling period for all participants as this will minimise the influence of seasonal changes in contacts to the GP clinic. It is a weakness that we do not have information on specific reasons for contact, that we have no information on socioeconomics and ethnicity, that we do not know who was responsible for registered email correspondence, and that different organisational structures of GP clinics may influence internal work distribution.

### Comparison with existing literature

Our population of 38 874 patients had contact to their general practice 7.9 times per year. This is slightly more than the 6–7 times, which has previously been reported,^12^ but including the 20% of patients in our population without contact to their GP clinic within the most recent year (9776 persons), we get an average annual contact of 6.3 times per year, which agrees with the national average. If we focus on the frequent attenders, they constitute 22% of the patient population and have on average 19.7 annual contacts to their general practice.

Our gross sample had a sex distribution like the Danish population (51.4% females), but frequent attenders are most often females (61.6%). This has been reported previously,^3,10^ and females generally are more often in contact with their GP clinic (Olsen J *et al*, unpublished data, 2025). Besides sex, the proportion of frequent attenders expectedly also increases with age and number of diagnoses.^6,8^ The general agreement between our findings and the literature renders support to the validity of our observations.

In our study sample and across all age groups, close to two out of three contacts were physical visits to the GP clinic. This is considerably higher than the 40% reported in a recent study of another group of Danish general practices (Olsen J *et al*, unpublished data, 2025) and may be explained by differences in the patient population (they included only patients aged >75 years), managerial policies, or because their sampling period included the COVID-19 pandemic with perhaps fewer physical visits.

In relation to a discussion on online illiteracy among older patients, it is worth noting that email consultations were relatively more frequent (21.1%) among the oldest group of patients (replaced phone and physical visits at the GP clinic) than among younger age groups. A caveat to this observation is, however, that we do not know whether it was the patient or a close relative or caretaker that actually was responsible for the mail correspondence. Another Danish study also observed that among a sample of 75-year-olds, 22% of their contact to the GP was by email (Olsen J *et al*, unpublished data, 2025). The Danish population generally has a high IT literacy with more than 95% of the population having access to the internet, which may explain these perhaps unexpectedly high numbers for email correspondence. A considerable proportion of the telephone and email contacts may be expected to be renewal of prescriptions and answers on previous clinical tests.

In contrast to previous studies, we had access to data on who was the contact in the general practice. Our data revealed that the GP would handle around 35.7% of all contacts and the staff in the clinic the other 64.3%. Compared with the clinical staff, a larger proportion of the patients handled by the GP are female, children, and the GP also appears to handle more of the patients that visit the GP clinic more seldomly. The latter makes good sense as this group of patients are often not well-known to the clinic. The same argument could also be valid for the children, where we see fewer contacts handled by email and relatively more by the GP. In contrast, but perhaps not surprising, the more frequent attenders are to a larger degree handled by the clinical staff.

### Implications for practice

The implication of a change towards more telephone or email contacts is the potential for a lower workload for GPs, but the premise is sufficient IT-literacy within the patient population.

Many of the daily contacts between patient and GP clinic (that is, blood pressure, annual checks for patients with chronic conditions such as diabetes) do not necessarily require a GP specialist and may equally well, without loss of quality, be handled by, for example, an experienced nurse. These observations are relevant to those countries and/or GP clinics with more limited use of clinical staff. There is an increasing focus on optimising resource allocations, and the current changes in demographics with an expected further increase in numbers of older patients may require a strong focus on the available staff resources in the GP clinic.

## Data

According to the ethical approval data sharing is not possible.
